# On Treatment of Convulsions in Infants

**Published:** 1876-07

**Authors:** 


### ARTICLE VIII.

### *On Treatment of Convulsions in Infants.*

Mons. Blanchez, in a lecture on diseases of children, in the *Medical Times and Gazette*, lays down the following rule for the treatment of convulsions. If it be a single attack, and gives no signs of tendency to recur, it is best to confine ourselves to some hygienic precautions, such as securing efficient ventilation, etc. If the attacks run into each other, or recur at short intervals, revulsives should be applied to the lower extremities, compresses of cold water, or of water with ether, being also laid on the temples. Compression may at the same time be made on the carotid arteries, as recommended by Trousseau. The pulsation of these vessels must be sought for at the lateral parts of the neck, and then they must be gradually compressed backward toward the spinal column. The amelioration should be rapid ; and if after two or three minutes it has not manifested itself in an evident manner, the compression should be longer continued. Inhalations of chloroform may then be resorted to, administering them in a very gentle and gradual manner. In order to avoid all danger, slight as this is, it is necessary that a certain quantity of air should be always mixed with the chloroform vapors. In some cases special indications present themselves, as for the employment of an emetic when it is well made out that the convulsions are due to indigestion. When the attack has been overcome, we must try to modify the general eclampsic condition by having recourse to anti-spasmodic treatment; but the management of agents of this description requires great prudence, several of them being of a dangerous character. Their dose is of great importance. For an infant from eight to fifteen months old, we should never exceed the dose of thirty centigrammes, after having commenced with five centigrammes. The maximum dose of belladonna powder is ten centigrammes, after commencing with one, increasing it very gradually, and carefully watching the throat and pupils of the child. We may proceed more boldly with oxide of zinc or James' powder (which M. Blanchez has not found of any special utility,) of which ten centigrammes may be given every two hours ; but bromide of potassium and chloral are to be preferred to any of these remedies. Of the bromide from ten to twenty centigrammes may be given every two hours, until fifty or sixty centigrammes are reached in an infant, and from two to three grammes in a child of seven. The effect should be manifest at the end of twenty-four hours, or the dose should be increased. A mixed treatment of the bromide and chloral gives little better results, the bromide being given during the day and the chloral at night. The maximum of the latter agent is twenty five centigrammes for an infant, and fifty for older children.—*Med. and Surg. Reporter.*

---

